# Isolation and Partial Characterization of Bioactive Fucoxanthin from *Himanthalia elongata* Brown Seaweed: A TLC-Based Approach

**DOI:** 10.1155/2013/802573

**Published:** 2013-05-09

**Authors:** Gaurav Rajauria, Nissreen Abu-Ghannam

**Affiliations:** School of Food Science and Environmental Health, College of Sciences and Health, Dublin Institute of Technology, Cathal Brugha Street, Dublin 1, Ireland

## Abstract

Seaweeds are important sources of carotenoids, and numerous studies have shown the beneficial effects of these pigments on human health. In the present study, *Himanthalia elongata* brown seaweed was extracted with a mixture of low polarity solvents, and the crude extract was separated using analytical thin-layer chromatography (TLC). The separated compounds were tested for their potential antioxidant capacity and antimicrobial activity against *Listeria monocytogenes* bacteria using TLC bioautography approach. For bio-autography, the coloured band on TLC chromatogram was visualized after spraying with DPPH and triphenyl-tetrazolium chloride reagents which screen antioxidant and antimicrobial compounds, respectively, and only one active compound was screened on the TLC plate. Preliminary identification of this active compound was done by comparing its colour and *R*_*f*_ (retention factor) value with the authentic fucoxanthin standard. Further, the active compound was purified using preparative TLC. This purified compound showed a strong antioxidant (EC_50_: 14.8 ± 1.27 *µ*g/mL) and antimicrobial (inhibition zone: 10.27 mm, 25 *µ*g compound/disc) activities, which were examined by DPPH scavenging and agar disc-diffusion bioassay, respectively. The bioactivity shown by the purified compound was almost similar to the fucoxanthin standard. The characteristic UV-visible and FT-IR spectra of the purified active compound completely matched with the standard. Hence, the main active compound in *H. elongata* was identified as fucoxanthin.

## 1. Introduction

Compared to terrestrial plants, seaweeds are an untapped resource offering substantial potential for the isolation of original natural ingredients of interest for food and health purposes. Of the diverse classes of seaweeds, edible brown seaweed is considered to be the most nutritious and possesses a range of compounds with biological properties [1]. The lipophilic fractions of these seaweeds are a mixture of components including carotenoid pigments especially fucoxanthin, zeaxanthin, violaxanthin, and other minor compounds such as *β*-carotene and anthocyanins derivatives [1]. Several reports have demonstrated the role of different carotenoids in the prevention of degenerative diseases, and this has been attributed to their antioxidant properties [2, 3]. However, some of these pigments are involved in cell communication and have been explored for their potential antimicrobial behaviour also [1, 4]. Therefore, these pigments play an important role in health maintenance and have traditionally attracted the attention of the pharmaceutical and food industry [5].

Among the pigments reported, fucoxanthin is one of the most abundant carotenoids, especially in brown seaweeds, and contributes >10% of the total estimated production of carotenoids in nature [6]. This pigment belongs to the group of xanthophyll, and recent reports suggest that fucoxanthin has several biological properties such as antioxidant, antimicrobial, antiobesity, and anticancer activities [7]. Previously, this pigment has been isolated and identified from edible brown seaweeds such as *Sargassum siliquastrum*, *Hizikia fusiformis,* and *Undaria pinnatifida* [8–10]. Though, these pigments can be easily assimilated, they cannot be synthesized by animal tissues; therefore, these compounds must be obtained from food [5]. Thus, there is an increasing need to find new sources and analytical procedure to screen and identify these molecules rapidly and precisely.

In this work, therefore, a rapid and reliable TLC-based approach was applied to isolate and purify the fucoxanthin pigments for the first time from *Himanthalia elongata* brown Irish seaweed. This approach includes the extraction of seaweed with previously optimized low polarity solvent mixtures, isolation of crude extract using analytical thin layer chromatography (TLC), biological screening of the extract for its antioxidant and antimicrobial activities using TLC bioautography, and purification of active compounds by preparative TLC. Furthermore, the purified compound was tested for its biological activities (*in vitro* assays) and chemically characterized by UV-visible and FT-IR spectroscopic techniques and compared with the authentic standard. To the best of our knowledge, this is the first time this compound has been biologically screened, isolated, and purified from *H. elongata* seaweed.

## 2. Material and Methods

### 2.1. Chemicals

For thin-layer chromatography analysis, HPLC Far UV gradient grade n-hexane, chloroform, diethyl ether, acetonitrile, acetic acid, and methanol were used (Merck Chemicals, Darmstadt, Germany). Triphenyl-tetrazolium chloride (TTC), 1,1-diphenyl-2-picrylhydrazyl (DPPH), potassium bromide (KBr), and fucoxanthin were purchased from Sigma-Aldrich Chemical Co. (Steinheim, Germany). 

### 2.2. Seaweed Sample and Extraction Procedure

Brown seaweed used in the present study was* Himanthalia elongata *which was purchased from Quality Sea Veg., Co Donegal, Ireland. Seaweed samples were thoroughly washed to remove epiphytes, sand, and debris and stored at –18°C until analysis. They were then crushed with liquid nitrogen and extracted according to the existing method in our laboratory [11] with equal-volume mixture of low polarity solvents (n-hexane, diethyl ether, and chloroform). All the dried extracts were dissolved in methanol and centrifuged at 9,168 ×g (Sigma 2–16 PK, Sartorius AG, Gottingen, Germany) for 10 min, and the supernatant was collected and used for further analysis.

### 2.3. Isolation and Purification of Active Compounds

#### 2.3.1. Analytical TLC

Analytical TLC was carried out on TLC plates (20 × 20 cm with 0.2 mm thickness, silica gel GF_254_, Merck, Darmstadt, Germany) cut from the commercially available sheets. An aliquot of crude extract was spotted onto the silica gel plate and allowed to dry for a few minutes. Afterwards, the plate was developed with chloroform/diethyl ether/n-hexane/acetic acid (10 : 3 : 1 : 1, v/v/v/v) as mobile phase in a previously saturated glass chamber with eluting solvents for 30 min at room temperature. The developed plate was dried under normal air and the spots were visualised under visible light. The *R*_*f*_ (retention factor) values of isolated compounds and standard were calculated and compared.

#### 2.3.2. TLC Bioautography for Bioactivity Screening

Bioautographic evaluation was conducted in order to check the antioxidant and antimicrobial activity of separated compounds on TLC plate. A fixed amount and concentration of extract (10 *μ*L of 10 mg/mL) was applied each time on the plate for TLC bioautography study. For the screening of antioxidant capacity, the developed air dried plate was sprayed with methanolic solution of 2.54 mM DPPH antioxidant reagent and the plates were air-dried after spraying. Bands with the antioxidant capacity were observed as yellow bands on purple background [2, 12].

For the screening of antimicrobial activity, bio-autographic evaluation of separated compounds was conducted using food pathogenic *Listeria monocytogenes *(ATCC 19115) bacteria as a test organism. Full-grown inoculums with a bacterial count of 1 × 10^6^ CFU/mL were prepared and centrifuged at 3000 ×g for 10 min. The supernatant medium was discarded and the pellet was redissolved in 10 mL fresh Mueller-Hinton broth (Scharlau Chemie, Barcelona, Spain). This culture was sprayed onto a developed TLC plate and incubated overnight at 37°C in 100% relative humidity. After incubation, the plate was sprayed with a 2% (w/v) solution of triphenyl-tetrazolium chloride (TTC) and incubated for further 6 h. Inhibition zone was observed as clear area against a red-coloured background on the TLC plate [13, 14]. 

#### 2.3.3. Preparative TLC for Purification

A streak of crude extract was applied manually on a preparative TLC glass plate (20 cm × 20 cm; 1500 *μ*m thickness) with inorganic fluorescent indicator binder (Analtech, Sigma-Aldrich, Steinheim, Germany). After air drying, the plate was developed, using the same mobile phase as used in the analytical TLC, in a presaturated glass chamber. In each experiment, two plates were used in parallel. One of the plates from each set of experiment was sprayed with DPPH radical (for antioxidants) and TTC solution (for antimicrobials), as described above, and the bands that showed antioxidant and antimicrobial activity were scraped off carefully from the second plate of each set of experiment. The scratched sample was dissolved in HPLC grade methanol and centrifuged at 12000 rpm for 15 min in order to remove silica. The supernatant was collected, filtered from 0.22 *μ*m filter, and dried under reduced pressure. Further, all the dried samples were passed under nitrogen gas for 5 min and then dissolved in methanol for further characterization and bioactivity analysis. The entire purification process was carried out under dark or dim light conditions.

### 2.4. Antioxidant and Antimicrobial Activities Determination (*In Vitro* Assay)

Antioxidant and antimicrobial activities of crude extract, purified compound, and standard were determined by DPPH radical scavenging capacity assay and disc diffusion bioassay, respectively, according to the methods reported earlier [11].

### 2.5. Characterization of Purified Compound

#### 2.5.1. UV-Visible Spectroscopy

The spectrum of the purified compound was recorded from 190 to 600 nm on a UV-visible spectrophotometer coupled with DAD detector (Agilent Technologies, Cork, Ireland). Peaks assignments were made by comparing the spectrum of analytes with fucoxanthin standard.

#### 2.5.2. FT-IR Spectroscopy

Fourier transform infrared (FT-IR) spectra of purified compound and fucoxanthin standard were recorded in KBr pellet using a Nicolet FT-IR spectrophotometer (AVATAR 360, Nicolet, Madison, WI, USA). Typically, 32 scans were signal-averaged for a single spectrum obtained within the region from 4000 to 500 cm^−1^. Dried sample or standard was mixed with dry KBr, and the mixture was pressed into a fine translucent disc. The sample was analyzed as KBr pellet and compared with the fucoxanthin standard.

## 3. Results and Discussion

### 3.1. Screening and Purification of Crude Extract

**Figure 1:**
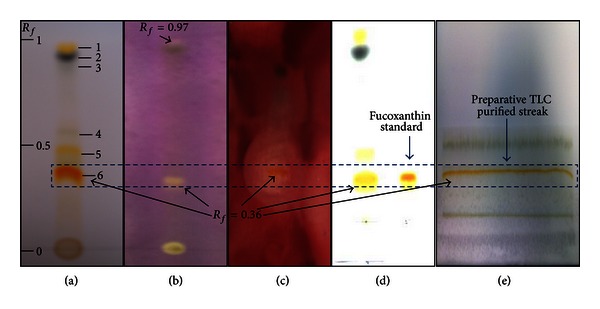
TLC based detection of compounds of *H. elongata* seaweed, visualized under visible light (a), bio-autographic screening of active antioxidant compound stained with 2.54 mM DPPH^•^ solution in methanol (b), bio-autographic screening of active antimicrobial compound stained with 2% aqueous TTC solution (c), identification of active compound with respect to fucoxanthin standard (d), and purification of active compound using preparative-TLC (e).

The present work describes a comprehensive methodology for the screening, purification, and characterization of bioactive carotenoid pigment from *H. elongata* seaweed. The extraction of seaweed was carried out by previously optimized, equal-volume mixture of n-hexane, diethyl ether, and chloroform solvents. The tested extraction solvents and their mixtures contained a wide range of polarity and the best crude extract was selected on the basis of its functional activities and total phenolic content (unpublished data). In this study, firstly, the compounds present in the selected crude extract were separated using analytical TLC. The chromatographic profile of the crude extract, visualized under visible light, indicated the presence of 6 colourful bands on the TLC plate. The main colourful pigments in *H. elongata* have been described as fucoxanthin, zeaxanthin, *β*-carotene, violaxanthin, echinenone, carotenoid, and chlorophyll [1, 4]. Thus, the orange, yellow, and green bands in TLC possibly would correspond to some of these pigments (Figure 1(a)). Analytical-TLC showed a strong yellow band at *R*_*f*_ = 0.97 (band 1) and light yellow bands (4 and 5) at *R*_*f*_ = 0.60 and 0.43, respectively. Moreover, TLC also exhibited greenish-black (bands 2, *R*_*f*_ = 0.93), greenish-grey (band 3, *R*_*f*_ = 0.87), and an intense orange colour band (6) at *R*_*f*_ = 0.36 (Figure 1(a)). Furthermore, the separated compounds were tested for their potential biological properties using TLC bioautography. For the antioxidant capacity, the developed plate was sprayed with DPPH^•^ reagent, and as it can be seen from the results (Figure 1(b)) that, of the separated compounds, only band 1 and 6 exhibited the antioxidant capacity as they turned yellow on purple background. The intensity of the yellow colour depends on the amount and nature of radical scavengers in the sample. The strength of the colour of band 6 was more intense as compared to the band 1 indicating that the former band had more antioxidant potential. It is reported that with the exception of fucoxanthin, other carotenoids such as *β*-carotene, *β*-cryptoxanthin, zeaxanthin, and lutein do not show scavenging effect against DPPH radical [15], which is in agreement with the present findings wherein other pigments did not show any scavenging against DPPH radical except the two compounds. DPPH^•^ is a commonly used substrate and has been widely used to screen antioxidant compounds from seaweed and other plants using TLC bioautography [2, 12, 16]. 

Regarding the antimicrobial activity, the developed TLC plate, preinoculated with *L. monocytogenes* culture, was sprayed with TTC agent, and a clear zone was observed (only at around band 6) on the TLC plate against red background (Figure 1(c)). Apart from band 6, none of the isolated compounds on TLC plate exhibited antimicrobial activity against *L. monocytogenes*. TLC bioautography-guided screening of antioxidant and antimicrobial activities of compounds in the crude extract is a quick approach and has been used by many researchers. For instance, antioxidant compounds in the crude extracts of *Spirulina platensis* alga and *Perilla frutescens* fruit were characterized by TLC bio-autography [2, 12]. Similarly, the antioxidant and antimicrobial activities of crude extracts of *Chlorococcum humicola* alga were determined using the same approach [16]. In this work, among the compounds isolated on the TLC plate, only band 6 showed antioxidant and antimicrobial properties and was considered the most active compound in *H. elongata* crude extract and, therefore, was analysed further.

Since fucoxanthin is the major carotenoid present in brown seaweed, it was used as a standard. In this regard, pure fucoxanthin along with the crude extract was simultaneously loaded on a single analytical TLC plate and its characteristic chromatographic profile was studied. Results from Figure 1(d) indicate that the intense orange colour and the R_f_ value of fucoxanthin standard are similar to one compound (band 6, *R*_*f*_ value: 0.36) isolated from the crude extract. Thus, the chromatographic identification suspected that the isolated active compound could be fucoxanthin. Hence, in order to check the authenticity, the active compound was purified using preparative TLC and a clear dark streak of an orange colour compound was separated from the crude extract and scratched from the plate (Figure 1(e)). Furthermore, this purified compound was tested for its potential antioxidant and antimicrobial activities using *in vitro* assays.

**Figure 2:**
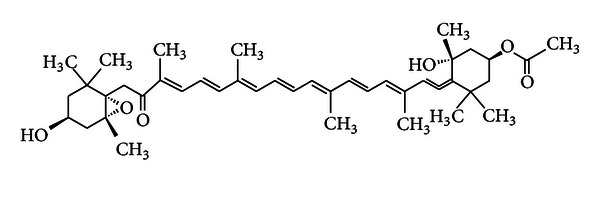
Chemical structure of fucoxanthin (molecular formula: C_42_H_58_O_6_).

The conventional spectrophotometric DPPH radical scavenging assay was used to screen the antioxidant capacity of the crude extract and preparative TLC purified compound of *H. elongata* seaweed. The crude extract exhibited lower scavenging capacity than the purified compound and standard. The DPPH radical was significantly scavenged by fucoxanthin standard and purified compound in a dose-dependent manner, with an EC_50_ value of 12.5 ± 0.98 *μ*g/mL and 14.8 ± 1.27 *μ*g/mL, respectively (Table 1). Therefore, it indicates that the compound isolated and purified from *H. elongata* seaweed showed almost similar antioxidant capacity to the standard of fucoxanthin. Despite the strong antioxidant nature of fucoxanthin, the chemistry behind the reaction between fucoxanthin and DPPH radical is unclear. However, some researchers anticipated that fucoxanthin reacts with equivalent mole of DPPH and donates an electron or a hydrogen radical under anoxic conditions to act as radical quencher. On the other hand, under aerobic conditions, fucoxanthin is partially oxidized by molecular oxygen and only a part of it reacts with DPPH rendering it relatively less active against the DPPH radicals [15]. The antioxidant capacity in carotenoids is closely related to the presence of intramolecular oxygen atoms and it appears to be more complex in case of fucoxanthin, having six oxygen atoms in the molecule (Figure 2), because antioxidants not only react with DPPH but also with oxygen [17].

**Figure 3:**
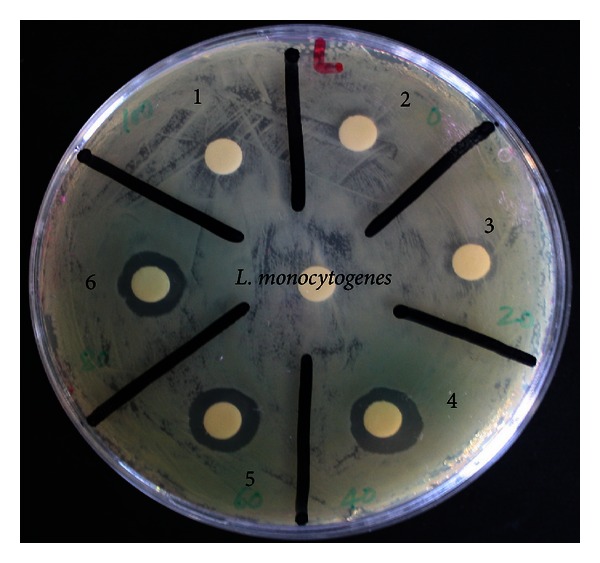
Antimicrobial activity of standard (fucoxanthin), crude extract, and purified compound against *L. monocytogenes* bacteria (control (1, 2, and 3); standard (4); purified compound (5); crude extract (6)).

Furthermore, the same purified compound was tested as an antimicrobial against *L. monocytogenes* bacteria using conventional disc diffusion bioassay. Twenty-five microlitres of crude extract, purified compound, and standard (fucoxanthin) was impregnated on the paper disc (Figure 3). The measurement of antimicrobial activity was based on the presence or absence of bacterial growth in the contact zone between the culture media and the samples and on the eventual appearance of an inhibition zone which was calculated as described in our previous publication [11]. The values of inhibition zone were recorded using digital vernier calliper (Draper ToolBox, Worcestershire, UK) with the average (mm) of two diameter measurements per disc taken in perpendicular directions. The inhibition zone of seaweed extract and purified compound was measured taking the reference of the inhibition exhibited by the standard and the results are illustrated in Table 1. The crude extract showed potent antimicrobial activity at a concentration of 10 mg/mL (250 *μ*g extract per disc) for *L. monocytogenes*. However, the activity shown by crude extract was much lower than the purified compound and the standard. The antimicrobial activity shown by both the purified compound and standard, at a concentration of 1 mg/mL (25 *μ*g/disc), was excellent and statistically similar (*P* < 0.05) against tested bacteria (Table 1). Therefore, it was evident from the previous results that the purified compound showed strong antioxidant and antimicrobial properties which were almost similar to the standard compound.

### 3.2. Characterization of the Purified Compound

**Figure 4:**
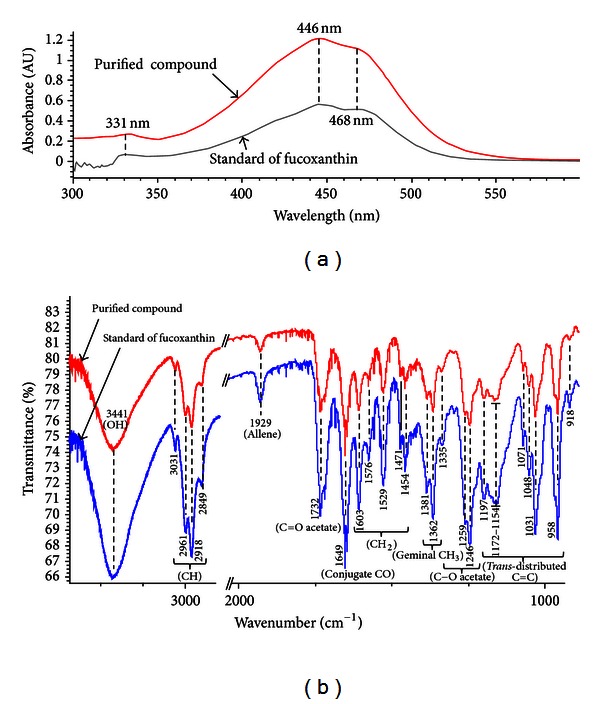
Characteristic UV-visible (a) and FT-IR (b) spectra of standard (fucoxanthin) and preparative TLC purified compound from *H. elongata* seaweed.

Preliminary identification done by TLC anticipated that the purified compound could be fucoxanthin. Furthermore, spectroscopic identification was conducted and compared with the authentic fucoxanthin standard to confirm the identity of the purified compound. In this regard, the UV-visible spectrum of the purified compound was recorded and its absorption maximum (*λ*_max⁡_) was compared with the fucoxanthin standard. Results can be seen from Figure 4(a) that both the standard and purified compounds exhibited the same spectroscopic profile with similar *λ*_max⁡_ (331, 446, and 468 nm). Fucoxanthin shows a characteristic absorption pattern (*λ*_max⁡_) in this region as reported by other researchers [1, 18]. Previously, the same compound has been identified in the crude extract of *H. elongata* and *Hizikia fusiformis* seaweed using UV-visible spectroscopy [4, 10]. 

In a subsequent experiment, the characteristic spectrum of the purified compound was recorded by FT-IR spectroscopy with the aim of attribution of the absorption bands that are characteristic of the functional groups present in the compound. It can be seen from Figure 4(b) that both the purified compound and fucoxanthin standard showed identical spectral fingerprint within the same region. The characteristic wave numbers of specific functional groups identified in both purified sample and standard were as follows: OH group (3441 cm^−1^), C–H stretch (3031–2849 cm^−1^), allene (1929 cm^−1^), C=O acetate (1732 cm^−1^), conjugated C=O (1649 cm^−1^), CH_2_ stretch (1603–1450 cm^−1^), geminal methyl (1381 and 1362 cm^−1^), C–O acetate (1335, 1259, and 1246 cm^−1^), and *trans*-distributed –C=C– (1201–958 cm^−1^), which agreed well with the reported data [19, 20]. Thus, cochromatography, UV-vis, and FT-IR data support that the major carotenoid in *H. elongata* is fucoxanthin.

## 4. Conclusion

In conclusion, TLC-guided approach (analytical, preparative, and bio-autographic) was used to screen and purify the bioactive compounds from *H. elongata* seaweed. One active compound with potential antioxidant and antimicrobial properties was identified as fucoxanthin. *H. elongata* may therefore be considered as a potential source of functional ingredients.

## Figures and Tables

**Table 1:** Antioxidant and antimicrobial activities of standard*, crude extract, and purified compound from *H. elongata* seaweed.

	Antioxidant capacity	Antimicrobial activity
	EC_50_ (*µ*g/mL)	Inhibition zone (mm)
Crude extract	91.3 ± 1.98^a^	9.95^a^
Purified compound	14.8 ± 1.27^b^	10.72^b^
Fucoxanthin*	12.5 ± 0.98^c^	10.89^b^

Values are expressed as average of three replicates.

Values with different letters (a–c) in each column are significantly different (*P* < 0.05).

Antioxidant capacity was determined using DPPH^*∙*^ scavenging assay.

Antimicrobial activity was determined against *L. monocytogenes* using disc diffusion bioassay. The diameter of the growth inhibition halos caused by the crude and purified samples and standard* was measured by a digital vernier caliper and expressed in millimetre.
